# First Detection and Characterization of *Smacovirus* in the Human Vagina in Two Sequential Samples over a Twelve-Day Interval

**DOI:** 10.3390/v16101545

**Published:** 2024-09-30

**Authors:** Antonio Charlys da Costa, Tania Regina Tozetto-Mendoza, Endrya do Socorro Foro Ramos, Pietro Bortoletto, Noely Evangelista Ferreira, Layla Honorato, Erick Matheus Garcia Barbosa, Heuder Gustavo Oliveira Paião, Amanda Fernandes de Souza, Iara M. Linhares, Steven D. Spandorfer, Elcio Leal, Maria Cassia Mendes-Correa, Steven S. Witkin

**Affiliations:** 1 Laboratorio de Virologia (LIM52), Departamento de Infectologia e Medicina Tropical, Instituto de Medicina Tropical de São Paulo, Faculdade de Medicina da Universidade de São Paulo, Av. Dr Enéas de Carvalho Aguiar, 470, São Paulo 05403-000, Brazil; charlysbr@yahoo.com.br (A.C.d.C.); noely.ferreira@gmail.com (N.E.F.); layla.honorato@usp.br (L.H.); erickmgb@usp.br (E.M.G.B.); heuder.paiao@gmail.com (H.G.O.P.); amandafernandessouza@usp.br (A.F.d.S.); maria.cassia@hc.fm.usp.br (M.C.M.-C.); switkin@med.cornell.edu (S.S.W.); 2 Laboratório de Diversidade Viral, Instituto de Ciências Biológicas, Universidade Federal do Pará, Belém 66075-110, Brazil; endrya.ramos@gmail.com (E.d.S.F.R.); elcioleal@gmail.com (E.L.); 3 Boston IVF-The Eugin Group, Waltham 02451, MA, USA; pietrobortolettomd@gmail.com; 4 Harvard Medical School, Boston 02115, MA, USA; 5 Department of Obstetrics and Gynecology, Beth Israel Deaconess Medical Center, Boston 02115, MA, USA; 6 Department of Gynecology and Obstetrics, Faculdade de Medicina da Universidade de São Paulo, São Paulo 05403-000, Brazil; iara.linhares@yahoo.com.br; 7 Center for Reproductive Medicine and Infertility, Weill Cornell Medicine, New York 10001, NY, USA; sdspando@med.cornell.edu; 8 Department of Obstetrics and Gynecology, Weill Cornell Medicine, New York 10001, NY, USA

**Keywords:** *Smacovirus*, vagina, genomic characterization, metagenomics

## Abstract

Background: *Smacovirus* is a CRESS-DNA virus identified almost exclusively in transient fecal samples from various vertebrate species. Objective: We evaluated human vaginal samples for the presence and maintenance of *Smacovirus*. Methods: Viral metagenomics analysis was performed on vaginal samples collected from 28 apparently healthy women in New York City, USA. Twenty-one of the women provided duplicate samples over a 12–21-day interval. Results: Phylogenetic analysis identified two samples from the same individual, collected over a twelve-day interval, that were positive for the complete *Smacovirus* genome. All detected sequence contigs belonged to a single variant of CRESS-DNA. Conclusions: The continuous presence of *Smacovirus* in the human vagina over a twelve-day period was identified.

## 1. Introduction

*Smacovirus*es are small, circular, Rep-Encoding Single-Stranded DNA (CRESS-DNA) viruses belonging to the *Smacoviridae* family [1]. To date, they have been detected by metagenomic analyses primarily in stool samples from a variety of vertebrates and insects [2]. It remains to be determined whether there are specific eukaryotic cell types and regions of the body capable of becoming infected by or harboring *Smacovirus*. Although *Smacovirus* has been suggested as a cause of diarrheal illnesses [2], it has also been identified in nonpathogenic conditions related to detection of human gut archaeal viruses [3,4]. CRISPR analysis has identified *Smacovirus* DNA sequences in the DNA of *Archaea*. This suggests that *Smacovirus*es might primarily or exclusively have a prokaryotic, rather than a eukaryotic, host [5].

We previously identified 11 near-full-length sequences of genomes belonging to species of CRESS-DNA viruses in the vaginas of healthy women undergoing cycles of in vitro fertilization in New York City [6]. This suggested that other CRESS-DNA viruses might also be present at this site. In the present communication, we report on the identification of two identical full-length complete sequences of *Smacovirus* in sequential vaginal samples obtained from one individual. Characteristics of the identified *Smacovirus* and details of its relationship to previously described *Smacovirus* isolates are delineated.

## 2. Materials and Methods

### 2.1. Population and Study Design

Participants in the present study were informed that the analysis aimed to investigate the presence of DNA viruses in vaginal secretions. Twenty-eight women agreed to participate and provided written informed consent. Their mean age was 36.6 years, ranging from 31 to 42. Among these participants, 21 women provided two separate vaginal samples, which were collected at intervals of 12 to 21 days between February and May 2022. A total convenience sample of forty-nine vaginal swabs was collected during their consultation at the Center for Reproductive Medicine and Infertility at Weill Cornell Medicine in New York City. Clinical and demographic data associated with the participants were retrieved through comprehensive chart review, which was conducted only after all laboratory analyses had been completed.

### 2.2. Sample Collection and Data

During a routine speculum-based examination, vaginal secretion samples were collected from the vaginal walls using a sterile cotton swab. To ensure thorough homogenization of the sample before transferring to the tube, the swab was vigorously shaken in a tube containing 1 mL of sterile phosphate-buffered saline. The samples were then stored at −80 °C. For analysis, the samples were shipped on dry ice to the Virology Laboratory (LIM-52) at the Faculdade de Medicina da Universidade de São Paulo (FMUSP) in São Paulo, Brazil. All vaginal samples were also screened for bacteria and fungi by metagenomics, as described previously [7].

### 2.3. Extraction, Enrichment of Viral DNA, and Library Preparation

For deep sequencing of the vaginal samples, a protocol for viral discovery and viral metagenomics was used, following previously published methods [6,8,9].

DNA extraction and purification were performed using the Maxwell^®^ RSC Viral Total Nucleic Acid Purification Kit (Promega, Madison, WI, USA), according to the manufacturer’s instructions.

For enrichment, a rolling circle amplification (RCA) protocol was employed using the TempliPhi 500 Amplification Kit (Cytiva, Marlborough, CA, USA), following the manufacturer’s instructions. The process involved combining 5 μL of the kit’s sample buffer with 1 μL of nucleic acid extract, heating the mixture to 95 °C for 3 min, and then cooling it in an ice-water bath. After cooling, 5 μL of reaction buffer and 0.2 μL of enzyme mix (Phi29 DNA polymerase) were added. The mixture was incubated at 30 °C for 24 h, followed by heat inactivation at 65 °C for 10 min.

Circular DNA was purified using the ProNex^®^ size-selective purification system (Promega, WI, USA) and quantified with the QuantiFluor^®^ ONE dsDNA System (Promega, WI, USA). Samples from each subject were treated with the Nextera XT Sample Preparation Kit (Illumina, San Diego, CA, USA) and used to build a DNA library, which was identified by a double barcode.

The library was purified using the ProNex^®^ size-selective purification system (Promega, WI, USA), and the amount in each sample was normalized to ensure equal representation of the library with the pooled samples using the ProNex^®^ NGS Library Quant Kit (Promega, WI, USA). For size range selection, Pippin Prep (Sage Science, Inc., Beverly, MA, USA) was used to select 600 bp (range: 500 to 700 bp), which excluded very short and long fragments.

The library was quantified again prior to cluster generation by qPCR using the ProNex^®^ NGS Library Quant Kit (Promega, WI, USA). In addition, it was sequenced in depth using a NovaSeq 6000 Sequencer (Illumina, CA, USA) with SP Reagent Kit v1.5, 500 cycles, and 2 × 250 bp ends (Illumina, CA, USA). The resulting metagenomic reads were trimmed, assembled de novo, and analyzed using an in-house pipeline [10,11].

### 2.4. Database and Viral Sequence Quality

Human host reads and bacterial reads were identified and removed by mapping the raw reads to human reference genome hg38 and bacterial genomes using bowtie2 in local search mode with other parameters set as default. We required approximately 60 bp aligned segments to be found, with at most 2 mismatches and no gaps [12]. Reads were considered duplicates if positions 5 to 55 from the 5 prime end were identical. One random copy of duplicates was kept. Low-sequencing-quality tails were trimmed using the Phred quality score 20 as a threshold. Adaptor and primer sequences were trimmed using the default parameters of VecScreen using default parameters [13].

We developed an ensemble strategy that integrated the sequential use of various de Bruijn graphs (DBGs) and overlap-layout-consensus assemblers (OLC) with a novel partitioned sub-assembly approach called Ensemble Assemble [10,11]; contigs and reads were both used for sequence similarity searches.

### 2.5. Alignment and Annotation

The assembled contigs and singlets were aligned to our viral proteome database using BLASTx (version 2.2.7) with an E-value cutoff of <0.01. Matches to viral proteins were then aligned to our non-redundant (NR) universal proteome database using DIAMOND version 2.1.4 [14] to filter out non-viral hits that had better alignments to non-viral species. Reference sequences were annotated as virus or non-virus according to NCBI taxonomy in the DIAMOND NR database. When a read or contig was searched against this database using DIAMOND, this read was assigned to virus or non-virus according to the best hit to the reference sequence with the lowest E-value.

### 2.6. Phylogenetic Reconstruction

Phylogenetic trees were constructed using the maximum likelihood approach, and branching support was estimated using an ultrafast bootstrap test with 1200 replications using the IQ-Tree tool [15]. Trees were visualized and edited using Figtree version 1.4.2 (http://tree.bio.ed.ac.uk/software/figtree, accessed on 1 February 2024). The tree depicts the evolutionary relationships among *Smacovirus*es, highlighting the main clades, such as human huchiSmacovirus 1, human huchiSmacovirus 2, and chicken huchiSmacoviruses.

## 3. Results

Only 4% (2/49) of the vaginal samples tested positive for *Smacovirus*. They were obtained from the same individual and were collected 12 days apart. The samples were from a 36-year-old white woman with a body mass index of 26.6 kg/m^2^. She was never pregnant, had no known infertility or underlying health problems, and was undergoing in vitro fertilization to obtain and freeze her oocytes to preserve her future fertility. Ten oocytes were obtained and frozen. The woman did not have diarrhea or any other current medical issues, and no other viruses were identified in either of her vaginal samples.

Both vaginal samples were negative for *Escherichia coli* and *Candida* species, microbes that are typically present in fecal samples and that can contaminate the vaginal microbiota. The predominant bacteria in both samples were *Lactobacillus crispatus*, *L. gasseri*, and *L. iners*.

**Figure 1:**
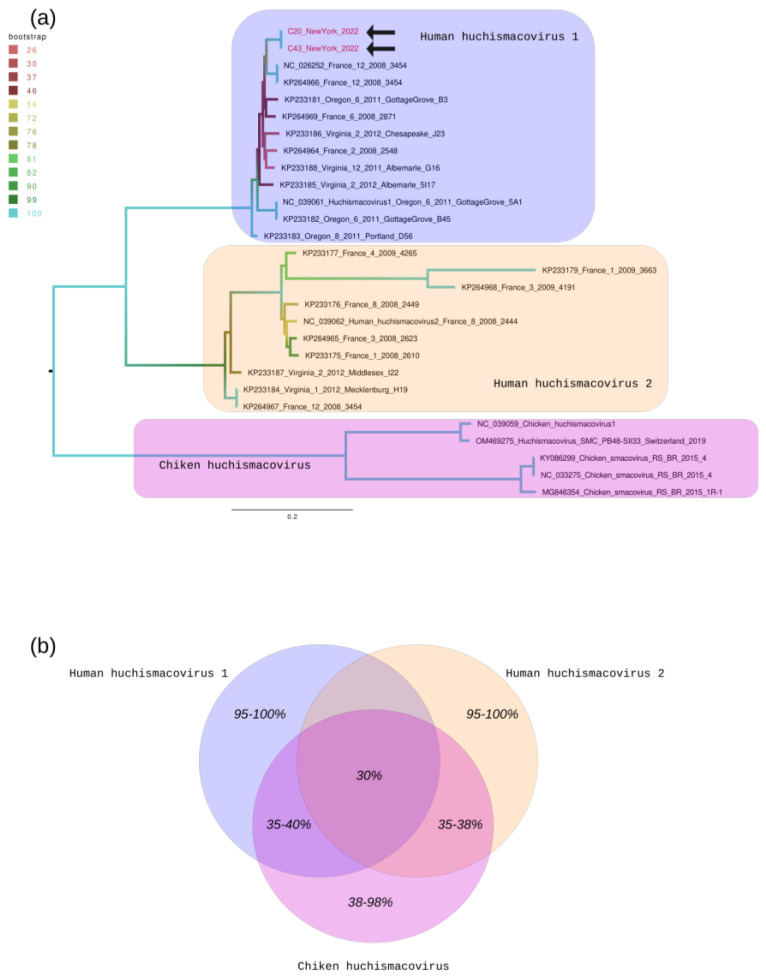
Genetic features of *Smacovirus*es. (**a**) Phylogenetic tree: The tree depicts the evolutionary relationships among *Smacovirus*es, highlighting the main clades such as human huchiSmacovirus 1, human huchiSmacovirus 2, and chicken huchiSmacoviruses. These clades are visually distinguished by different-colored areas within the tree. Arrows within the human huchiSmacovirus 1 clade represent sequences from this study. Branch colors indicate the bootstrap statistical support, as indicated by the scale provided in the upper left corner of the tree. Branch lengths are scaled in nucleotide substitutions per site, as per the scale below the tree. (**b**) Venn diagram: The diagram illustrates the nucleotide similarity among genomes of *Smacovirus*es. It compares sequences within each clade and also between the clades, providing insight into genetic relationships within and across different *Smacovirus* groups. The *Smacovirus* isolates have been deposited in GenBank under accession number PP542036.

We identified two sequences-contigs that represented the complete *Smacovirus* genome. The orthology of the *Smacovirus* DNA sequences was confirmed through blast N and BlastX searches. Genomic structural information of the *Smacovirus* sequences (GenBank under accession number PP542036) can be seen in Table 1. Phylogenetic analysis revealed that all sequences detected belong to a single clade, which included *Smacovirus* 1 references (Figure 1). Clade samples previously identified from France and Oregon in the US exhibited robust bootstrap support, exceeding >80% [2]. Both of the samples in the present study exhibited robust bootstrap support, exceeding >99%.

## 4. Discussion

*Smacovirus* was identified in two successive samples taken from the vagina of an apparently healthy woman. The presence of *Smacovirus* in the human vagina has not been described previously. Its continued identification in two sequential samples obtained over a twelve-day time period is longer than what has previously been described in the literature for eukaryotic-derived samples [2]. In an earlier study, we unsuccessfully attempted to establish a small-animal model for human stool-associated *Smacovirus*es by inoculating fecal filtrates into the gastrointestinal tract of an adult immunodeficient mouse by gavage feeding. However, no viral DNA was detected in stool samples by gene amplification after day 2 post-inoculation. Similarly, after oral inoculation of four mice with a human *Smacovirus*-positive stool-derived sample, all internal organs remained negative for this virus, except for the cecum, which was positive only for two days [2]. These preliminary findings were consistent with the possibility that the human-stool-associated *Smacovirus* was transited to the gut and passively shed in the stools without invasion or replication at sites within the body [2].

Possible contamination of the vaginal microbiota by microorganisms originating in the rectum has been detailed [16]. Thus, *Smacovirus* in the vagina might simply indicate rectal contamination. An alternate explanation for our observation of *Smacovirus* in the vagina and its maintenance for at least 12 days is that this virus was a component of the vaginal econiche and was not merely a fecal contaminant. The vaginal bacterial and fungal microbiomes in the woman positive for vaginal *Smacovirus* were typical of those of fertile women, and both samples were negative for microorganisms typical of fecal contamination. The absence of *E. coli* and *Candida fungi* suggests, but does not definitely exclude, that the *Smacovirus* detected in vaginal samples on two separate occasions was not merely a fecal contaminant. In addition, the presence of *Smacovirus* in the vagina in the present study was not associated with any detectable disturbance of the vaginal microbiota.

The identity of the cells type(s) that may have been infected by *Smacovirus* in this woman remains unknown. Possibilities include epithelial cells, leukocytes, or bacterial genera that are present in the vagina. Alternatively, their presence may be the result of its transduction from the circulation into the vagina. Unfortunately, in the present study, serum or fecal material from this woman was not available for analysis to further explore these possibilities.

In conclusion, the complete *Smacovirus* genome was identified in two consecutive vaginal samples from the same women. This provides the first evidence that this virus can be continuously present for at least a twelve-day time period in humans at a location outside of the colon.

## Figures and Tables

**Table 1:** Characterization of *Smacovirus* isolates from consecutive vaginal samples obtained from a single individual.

	Sample 1	Sample 2
Date of collection	15 February 2022	26 February 2022
Length of fragment (bp)	2466	2466
Coverage	2350×	1560×
Number of reads	23,180	15,289
^a^ Genbank prototype	KP264966	KP264966
Country	France	France
Percent identity	96%	96%
Year identified	2008	2008

Samples 1 and 2 were deposited in Genbank under accession number PP542036. Base pairs: bp. ^a^ The access number denotes the prototype sequence most closely related to the one identified in this study.

## Data Availability

Data are available via the corresponding author upon reasonable request.

## References

[1] Varsani A., Krupovic M. (2018). Smacoviridae: A new family of animal-associated single-stranded DNA viruses. Arch. Virol..

[2] Ng T.F.F., Zhang W., Sachsenröder J., Kondov N.O., da Costa A.C., Vega E., Holtz L.R., Wu G., Wang D., Stine C.O. (2015). A diverse group of small circular ssDNA viral genomes in human and non-human primate stools. Virus Evol..

[3] Chibani C.M., Mahnert A., Borrel G., Almeida A., Werner A., Brugère J.-F., Gribaldo S., Finn R.D., Schmitz R.A., Moissl-Eichinger C. (2021). A catalogue of 1167 genomes from the human gut archaeome. Nat. Microbiol..

[4] Gregory A.C., Zablocki O., Zayed A.A., Howell A., Bolduc B., Sullivan M.B. (2020). The Gut Virome Database Reveals Age-Dependent Patterns of Virome Diversity in the Human Gut. Cell Host Microbe.

[5] Díez-Villaseñor C., Rodriguez-Valera F. (2019). CRISPR analysis suggests that small circular single-stranded DNA smacoviruses infect Archaea instead of humans. Nat. Commun..

[6] Ramos E.d.S.F., Tozetto-Mendoza T.R., Bortoletto P., Ferreira N.E., Honorato L., Barbosa E.M.G., Luchs A., Linhares I.M., Spandorfer S.D., Leal E. (2024). Characterization of CRESS-DNA viruses in human vaginal secretions: An exploratory metagenomic investigation. J. Med. Virol..

[7] Da Costa A.C., Bortoletto P., Spandorfer S.D., Tozetto-Mendoza T.R., Linhares I.M., Mendes-Correa M.C., Witkin S.S. (2023). Association between torquetenovirus in vaginal secretions and infertility: An exploratory metagenomic analysis. Am. J. Reprod. Immunol..

[8] da Costa A.C., Moron A.F., Forney L.J., Linhares I.M., Sabino E., Costa S.F., Mendes-Correa M.C., Witkin S.S. (2021). Identification of bacteriophages in the vagina of pregnant women: A descriptive study. BJOG Int. J. Obstet. Gynaecol..

[9] Ramos E.d.S.F., Rosa U.A., Ribeiro G.d.O., Villanova F., Milagres F.A.d.P., Brustulin R., Morais V.d.S., Araújo E.L.L., Pandey R.P., Raj V.S. (2021). Multiple clades of Husavirus in South America revealed by next generation sequencing. PLoS ONE.

[10] Deng X., Naccache S.N., Ng T., Federman S., Li L., Chiu C.Y., Delwart E.L. (2015). An ensemble strategy that significantly improves de novo assembly of microbial genomes from metagenomic next-generation sequencing data. Nucleic Acids Res..

[11] Langmead B., Salzberg S.L. (2012). Fast gapped-read alignment with Bowtie 2. Nat. Methods.

[12] Ye J., McGinnis S., Madden T.L. (2006). BLAST: Improvements for better sequence analysis. Nucleic Acids Res..

[13] Altan E., Delaney M.A., Colegrove K.M., Spraker T.R., Wheeler E.A., Deng X., Li Y., Gulland F.M.D., Delwart E. (2020). Complex Virome in a Mesenteric Lymph Node from a Californian Sea Lion (*Zalophus californianus)* with Polyserositis and Steatitis. Viruses.

[14] Buchfink B., Reuter K., Drost H.-G. (2021). Sensitive protein alignments at tree-of-life scale using DIAMOND. Nat. Methods.

[15] Trifinopoulos J., Nguyen L.-T., von Haeseler A., Minh B.Q. (2016). W-IQ-TREE: A fast online phylogenetic tool for maximum likelihood analysis. Nucleic Acids Res..

[16] El Aila N.A., Tency I., Claeys G., Verstraelen H., Saerens B., Santiago G.L.d.S., De Backer E., Cools P., Temmerman M., Verhelst R. (2009). Identification and genotyping of bacteria from paired vaginal and rectal samples from pregnant women indicates similarity between vaginal and rectal microflora. BMC Infect. Dis..

